# Digital Business in der Praxis: Modell, Analyse und Handlungsfelder

**DOI:** 10.1007/978-3-658-32323-3_1

**Published:** 2021-03-06

**Authors:** Kim Oliver Tokarski, Ingrid Kissling-Näf, Jochen Schellinger

**Affiliations:** 1 Berner Fachhochschule Wirtschaft, Bern, Switzerland; 2 Berner Fachhochschule Wirtschaft, Bern, Switzerland; 3 Berner Fachhochschule Wirtschaft, Bern, Switzerland; Berner Fachhochschule Wirtschaft, Bern, Schweiz

## Abstract

Der Beitrag beschäftigt sich mit der digitalen Transformation von Organisationen. Er zeigt auf der Basis der Beiträge dieses Sammelbands und der angeführten Ergebnisse eigener empirischer Studien, welche Veränderungsprozesse in welcher Tiefe und in welchem Feld beobachtet werden können. Anhand eines maturitätsorientierten Analysemodells zum digitalen Business werden die Transformationsprozesse in den einzelnen Fallstudien zuordenbar. Die Beiträge dieses Sammelbandes reflektieren die Annahme, dass sich die digitale Transformation in ihren unterschiedlichen Ausprägungen grundsätzlich intensiviert. Besonders deutlich wird dies für Prozesse der Automatisierung in der Industrie und auch für den Begleitprozess im Bereich Human Resources, die den Change strategisch begleiten und in einer neuen Art unterstützen.

## Das Digitale als Treiber für den Umbau zur postindustriellen Gesellschaft

„Das Digitale ist der Treiber der vierten industriellen Revolution. Das Digitale ist aber nicht nur Technik – es ist eine neue Art, Unternehmen zu führen“ (Sprenger 2018, S. 20). So weist Reinhard Sprenger zu Recht darauf hin, dass es um Integration des Digitalen in jedwede Geschäftstätigkeit geht, sei es Produktion, Vertrieb, Marketing, Finanzen oder Human Resource Management (HRM).

Der Umbau zu einer postindustriellen Gesellschaft begann in den achtziger Jahren des 20. Jahrhunderts. Der Industriesektor nahm zugunsten der Dienstleistungen und der Wissensökonomie ab. Der damit verbundene Dynamisierungsschub ist, wie die Finanzkrise, die Migrationskrise und die Corona-Krise zeigen, um einiges stärker als die durch die Industrialisierung ausgelösten Entwicklungen nach dem Zweiten Weltkrieg, die regulierter und stärker am Nationalstaat orientiert waren. Der liberale Staat selbst hat die Entwicklungsdynamiken mit dem globalen Handel und der Deregulierung weiter vorangetrieben und sieht sich nun verpflichtet, resilienter zu werden und sich für öffentliche Güter wie Gesundheit und Klima wieder vermehrt einzusetzen bzw. Katastrophen und Pandemien zu verhindern (Interview mit A. Reckwitz 2020, S. 23).

Wie uns COVID-19 gezeigt hat, kommen Veränderungen über Nacht. Besonders deutlich wurde dies beim Homeoffice, dem Onlinemarketing oder auch der Telemedizin, die sich in der Pandemiephase bewährten und eine gewisse neue Normalität für die Arbeitswelt, die Versorgung mit Lebensmitteln und medizinischer Leistung in der Pandemie ermöglichten. Ohne entsprechende digitale Tools hätten Unternehmen kaum so agil auf die Pandemie reagieren können. Verschiedene Studien (z. B. Gurtner und Hietschold 2020) zeigen, dass jene Unternehmen sich in der COVID-19-Krise besser geschlagen haben, die eine gut ausgebaute digitale Kommunikationsinfrastruktur wie auch eine entsprechende Unternehmenskultur besitzen und ihren Mitarbeitenden flexible Arbeitsmodelle anbieten und bereit sind, sich kurzfristig anzupassen.

Dem dritten Digital-Vortex-Bericht 2019 des International Institute for Management Development (IMD) ist zudem zu entnehmen, dass weiterhin speziell fünf Branchen seit 2015 der digitalen Disruption stark unterworfen bleiben: Es sind dies die Medien- und Unterhaltungsindustrie, die technologischen Produkte und Dienstleistungen, die Telekommunikationsbranche, der Detailhandel und die finanziellen Dienstleistungen. Speziell die Vulnerabilität der Transport- und Logistikbranche scheint maßgeblich anzusteigen. Die Daten des IMD zeigen auch, dass der Anteil der Unternehmen, die über eine digitale Strategie verfügen, von 54 % auf 75 % steigt, jedoch nur 22 % über eine sogenannte koordinierte digitale Strategie verfügen.

Hier wiederum kann nochmals an Sprenger angeknüpft werden, der auf die Dynamik der digitalen Transformation hinweist, zugleich aber immer auch betont, dass das, was als technologische Revolution beschrieben wird, eigentlich die „Wiedereinführung des Menschen in die Unternehmen“ meint (Sprenger 2018). So ist im Rahmen der digitalen Transformation eine Konzentration auf die „K’s“ zentral: Damit gemeint ist die Wiedereinführung des Kunden, der Kooperation und der Kreativität.

Während Unternehmen sich in den vergangenen Jahrzehnten zu stark auf sich konzentriert haben (Innenfokussierung), verlangt die digitale Transformation, das ganze Unternehmen wieder vom Kunden her zu denken (Außenfokussierung). Zudem haben Spezialisierung und Expertentum dazu geführt, dass die Aufgaben „zerteilt“ wurden. Digitalisierung hingegen fordert von den Arbeitnehmenden neue Formen der Zusammenarbeit, welche funktions- und hierarchieübergreifend, interdisziplinär und unternehmensübergreifend funktionieren. Die effiziente Produktion hat des Weiteren die Kreativität verdrängt, bzw. Letztere wurde ausgelagert in Labore (Labs), Inkubatoren, Start-ups und Impact Hubs. Informationen, Forschung und Design gewinnen jedoch an Bedeutung und damit auch das kreative Schaffen, das für das Überleben von Industrien zentral wird.

Die digitale Transformation ist damit nicht technologisch zu bewältigen, sondern stellt den Kulturprozess und den Menschen ins Zentrum. Die Kunst der Führungskraft ist es nicht mehr, individuell Arbeitnehmende zu motivieren und personenzentriert zu agieren, sondern vielmehr zu überlegen, ob die Unternehmung als Organisation richtig aufgestellt ist, damit sie mit der digitalen Welt kompatibel ist: Alt und neu sind zu verbinden, neue Komplexität ist ins laufende System einzubauen, neue Geschäftsmodelle sind mit der Organisation zu verbinden; oder anders formuliert: Der Weg geht von der Fehlervermeidung zum Ausprobieren, von der Vorgabe zur Selbstverantwortung, von der Binnenorientierung zur Außenorientierung.

Einblick darüber, wie diese technologisch induzierten Veränderungen in Unternehmen, Politik sowie in der Gesellschaft ablaufen, wo sie starten, welchen Verlauf sie nehmen und wie sie unterstützt und analysiert werden können, wird in den Beiträgen des vorliegenden Sammelbands gewährt. Den Ausgangspunkt bildet ein Analysemodell für Digital Business und digitale Transformation, das ausgehend von der kundenzentrierten Leistungserstellung die zentralen Bausteine des organisationalen Wirkens aufnimmt und die Maturitätsstufen der digitalen Transformation abbildet, um anschließend die verschiedenen Studien und Fallbeispiele einzuordnen.

## Ein Analysemodell für Digital Business und digitale Transformation

Das vorliegende Herausgeberwerk beschäftigt sich mit dem Digital Business verstanden als digitale Transformation von Organisationen, speziell im Wirtschaftskontext. Doch was ist hierunter genau zu verstehen? Was beinhaltet die digitale Transformation von Organisationen? Wie kann diese realisiert werden? Welche (Maturitäts-)Stufen gibt es in diesem Zusammenhang? Um diese Fragen klären zu können, wird das in Abb. 1.1 dargestellte Transformationsmodell vorgeschlagen.

**Figure 1:**
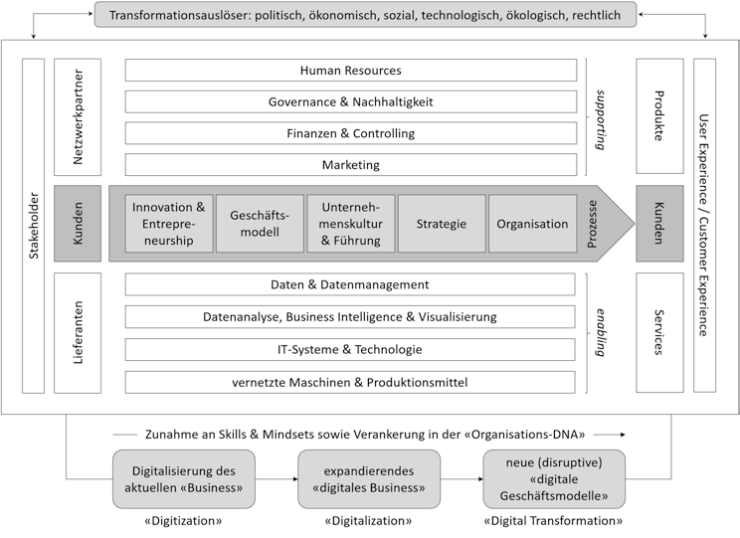

Im Rahmen der Organisationsentwicklung und somit auch im Kontext des Digital Business ergeben sich für das Unternehmen von außen betrachtet aus der Unternehmensumwelt unterschiedliche (mögliche) Transformationsauslöser. Diese können auf Basis des PESTEL-Modells in der deutschsprachigen Übersetzung als politische, ökonomische, soziale, technologische, ökologische und rechtliche Faktoren gesehen werden (siehe hierzu beispielsweise Schomaker und Sitter 2020 oder Yüksel 2012). Je nachdem, wie die Wahrnehmung dieser Faktoren im Unternehmen durch die Mitarbeitenden und Führungskräfte im Sinne von Einstellungen und Mindsets ausgestaltet sind, können Veränderungen aus der Unternehmensumwelt als Chancen bzw. Gelegenheiten oder Risiken wahrgenommen respektive bewertet werden. Wahrgenommene Transformationsauslöser werden letztlich durch die Organisationsmitglieder im Unternehmen in Form von Prozessen „verarbeitet“. Dem Prozess kommt im Modell somit eine wesentliche Bedeutung zu.

Den Kern des Modells bilden zentrale, kundenorientierte End-to-End-Leistungserstellungsprozesse der Organisation. Die Prozesse sind als Bindeglied der betriebswirtschaftlichen Funktionen vom Kunden zum Kunden („end-to-end“) zu verstehen. End-to-End bedeutet somit hierbei, dass von den Bedürfnissen und Anforderungen der Kunden aus die Leistung der Organisation gedacht und letztendlich in Form von Produkten oder Dienstleistungen (Services) erbracht wird. Dabei kommt der Leistungserbringung in Form von „User Experience“ bzw. „Customer Experience“ eine zentrale Bedeutung zu. Die User Experience beinhaltet im Kern u. a. Aspekte der Usability sowie des „Look“ und des „Feel“. Eine zielgerichtete User Experience ergibt sich in der Verbindung von Bedürfnissen des Kunden sowie wirtschaftlichen Aspekten, als auch technologischen Aspekten eines Produktes, bzw. einer Dienstleistung. Aufbauend hierauf kann die Customer Experience noch etwas weitergehend verstanden werden, da diese beispielsweise Service-, Branding- und Preisaspekte sowie den Verkaufsprozess in der Interaktion mit dem Kunden betrachtet. Im Kern geht es bei beiden Konzepten darum, ein positives Erlebnis mit „bedürfnisbefriedigenden“ Produkten, bzw. Dienstleistungen für die Kunden zu schaffen und dabei eine gute, nachhaltige Interaktion zu erzeugen. Speziell im Rahmen der digitalen Transformation kommt diesen Konzepten eine besondere Bedeutung zu, da hier eine hohe (digitale) Interaktion mit den Kunden aufzufinden ist. Zum Ende soll die Verwendung von Produkten, Dienstleistungen und die Interaktion mit dem Unternehmen dem Kunden „Freude“ („joy of use“) bereiten. Doch wie werden kundenzentrierte Produkte und Dienstleistungen im End-to-End-Leistungserstellungsprozess erzeugt? Welche grundlegenden Bestandteile sind hierzu nötig?

Als ein wesentlicher Startpunkt wird der Bereich „Innovation und Entrepreneurship“ gesehen. Dieser bildet den Ausgangspunkt der Leistungserstellungsprozesse. So ist Entrepreneurship eine Denk- und Handlungsweise, welche u. a. die Kundin bzw. den Kunden ins Zentrum der Betrachtung stellt. Veränderungen, innerhalb des Unternehmens oder von außen aus der Umwelt, werden positiv als Chance bzw. Gelegenheit (Opportunity) betrachtet. Dabei kommt dem Bereich der „Opportunity Recognition“, also der Wahrnehmung von Chancen bzw. Gelegenheiten und deren Nutzung, eine besondere Bedeutung zu. Im Entrepreneurship geht es dabei auch um die (neue) Kombination von Ressourcen, um sich bietenden Veränderungen wirtschaftlich zu nutzen (siehe hierzu etwa Volkmann und Tokarski 2006; Volkmann et al. 2010). Wenn sich eine Veränderung ergibt, stellen sich im Entrepreneurship zentrale, positive und gestaltende Fragen, wie zum Beispiel:

- Wie kann eine Veränderung genutzt werden?
- Was kann damit gemacht werden?
- Welche neuen Möglichkeiten ergeben sich hieraus?
- Was ist zu tun?

Diese Fragen und diese Denkhaltung sind eng mit dem Bereich der Innovation verbunden, denn im Kontext der Innovation geht es um die Erstellung, das „an den Markt bringen“ und die Diffusion (Durchsetzung und Bewährung am Markt) von neuen Produkten oder Dienstleistungen (siehe zur Diffusion von Innovationen grundlegend Rogers 2003 sowie kritisch hierzu Lundblad 2003). Der Neuheitsaspekt kann dabei als „absolut“ neu oder „relativ“ neu (für einen Markt oder ein Unternehmen) betrachtet werden (z. B. Dropbox, Google Drive oder OneDrive). Im Rahmen der wirtschaftlichen Tätigkeit und speziell vor dem Hintergrund der digitalen Transformation ergeben sich somit neue Produkte und Dienstleistungen, welche stark mit dem Geschäftsmodell des Unternehmens verbunden sind.

Das Geschäftsmodell ist die abstrakte Darstellung der wirtschaftlichen Tätigkeiten eines Unternehmens auf einem hohen Abstraktionsniveau. Der Definition von Gassmann et al. (2014) folgend, beinhaltet das Geschäftsmodell drei grundlegende Bereiche: (1) Nutzenversprechen (Was bieten wir den Kunden an?), (2) Wertschöpfungskette (Wie stellen wir die Leistung her?) sowie (3) Ertragsmechanik (Wie wird Wert erzielt?). Im Kern des Geschäftsmodells stehen dabei die Kunden (Wer sind unsere Kunden?). Zusammenfassend liefert das Geschäftsmodell Antworten auf die zuvor aufgeführten Fragen und die zentrale Frage: Was tut das Unternehmen im Kern?

Hiermit ist auch die normative Ebene der Unternehmenskultur und Führung der Unternehmung verbunden. Wie bereits erwähnt ist Entrepreneurship eine Denk- und Handlungsweise. Als Denkweise ist sie ein Bestandteil der Unternehmenskultur oder auch gerade nicht, wenn die Unternehmenskultur beispielsweise eher starr, bürokratisch, hierarchisch, unflexibel oder nicht offen gegenüber neuen Ideen ist. Speziell im Rahmen der digitalen Transformation bzw. des Digital Business braucht es eine unternehmerische Denk- und Handlungsweise, um die sich bietenden Chancen (schnell) nutzen und in neue Geschäftsmodelle sowie Produkte und Dienstleistungen transformieren zu können. Die Aufgabe der Führungskräfte ist die Förderung einer solchen Ausrichtung des Unternehmens. Bei Start-ups bzw. jungen Unternehmen wie etwa Airbnb, Spotify, Wish, Pinterest, instacart, Lunaphore, Versantis etc. ist dies „einfacher“ der Fall, da Entrepreneurship ein Teil der „Organisations-DNA“ ist. Etablierte Unternehmen und speziell Großunternehmen tun sich hier oft etwas schwerer, da sie eine ausdifferenzierte Unternehmenskultur haben, welche oft in einem Spannungsverhältnis zwischen Kosten und Innovation steht. Die Kunst ist es, beide Bereiche innerhalb eines Unternehmens zu vereinen. Hierbei wird vom Konzept der „Ambidexterity“, der Beidhändigkeit, gesprochen (siehe zur Ambidexterity grundlegend O’Reilly III und Tushman 2013). Im Rahmen der digitalen Transformation ist ganz speziell auf eine Transformation der Unternehmenskultur und auch der Führung hinzuwirken.

Aufbauend auf den zuvor dargestellten Bereichen ergibt sich die spezifische Ausrichtung des Unternehmens durch die Strategie. Eine Strategie kann verstanden werden als Planung der Ziel-Maßnahmen-Kombinationen (Was wollen wir tun? Wie erreichen wir dies?). Im Rahmen der Strategie ist es wichtig zu wissen und zu planen, was getan werden soll. Gleichermaßen ist es auch wichtig zu wissen, was nicht getan werden soll, denn eine Strategie ist immer vor dem Hintergrund beschränkter Ressourcen (z. B. Zeit, Finanzen, Arbeitskräfte, etc.) zu betrachten.

Zum Ende sind alle zuvor beschriebenen Bereiche (Innovation und Entrepreneurship, Geschäftsmodell, Unternehmenskultur und Führung, Strategie) operativ in Form von Organisation umzusetzen und in explizite oder implizite Prozesse zu überführen. Die Organisation, egal ob „klassisch“ hierarchisch, als Prozessorganisation oder in Form von Selbstorganisationsansätzen, realisiert zum Ende die Leistungserstellung und „transformiert“ normative und strategische Aspekte im Unternehmen bzw. einer Organisation.

Unterstützt und mit umgesetzt wird der Leistungserstellungsprozess durch indirekte Leistungsbereiche wie Human Resource Management, Governance und Nachhaltigkeit, Finanzen und Controlling oder das Marketing.

Was ist nun aber das Neue im Bereich der digitalen Transformation? Es ist der Umfang und die Geschwindigkeit mit dem Unternehmen auf Transformationsauslöser reagieren (müssen) und die Organisationsentwicklung vorantreiben. Als „enabler“ wirken dabei u. a. Daten, Datenmanagement, Datenanalyse, Business Intelligence und Visualisierung (oft über Unternehmensgrenzen hinweg), vernetzte IT-Systeme, Maschinen und Produktionsmittel. Im Kern erfolgt eine umfassende Nutzung digitaler Technologien im Rahmen der Digitalisierung, z. B. in Form von Automatisierung und Vernetzung in und zwischen Unternehmen und ihren Stakeholdern (z. B. Lieferanten, Kunden, Staat, etc.).

Vor diesem Hintergrund können grundlegend drei Stufen der Transformation im Digital Business definiert werden. In der ersten Stufe geht es um die Digitalisierung des aktuellen „Business“. Analoge Instrumente und beispielsweise Prozesse werden digitalisiert. Im Englischen wird von „Digitization“ gesprochen. Die zweite Stufe umfasst ein expandierendes „digitales Business“. Instrumente, Methoden und Prozesse werden „weitergedacht“ und genutzt. Dies wird im Englischen als „Digitalization“ bezeichnet. In der dritten Stufe werden auf Basis neuer Technologien, Daten, Methoden und Instrumente neue (disruptive) „digitale Geschäftsmodelle“ entwickelt, kombiniert und mit weitreichenden Konsequenzen am Markt und auch in der Gesellschaft umgesetzt. Im Englischen wird dies als „Digital Transformation“ beschrieben. Von der Stufe 1 über die Stufe 2 hin zur Stufe 3 kann u. a. eine Zunahme von Skills und Mindsets sowie eine stärkere Verankerung in der „Organisations-DNA“ festgestellt werden.

Das vorgestellte Modell liefert eine spezifische, wenngleich nicht abschließende Sichtweise auf die Thematik der digitalen Transformation. Es dient als Erklärungsansatz der Wechselwirkung zwischen den Transformationsauslösern und deren prozessualer Nutzung im Rahmen der Unternehmung. Weiterhin verdeutlicht es drei Maturitätsstufen im Kontext der digitalen Transformation. In einer Gesamtbetrachtung erklärt das Modell das Was, aber noch nicht das Wie der digitalen Transformation. Letzteres wird in Form von Beispielen und Fallstudien aus der Praxis für die Themenbereiche durch die Kapitel des vorliegenden Herausgeberwerkes realisiert. Auf Basis des Modells soll dabei u. a. auch eine bessere Einordnung in einen übergeordneten Gesamtkontext ermöglicht werden.

## Anwendungsbeispiele in der Praxis

Die ersten drei Beiträge des Sammelbandes befassen sich schwerpunktmäßig mit Rahmensetzungen und technologischen Aspekten der digitalen Transformation im ökonomischen Kontext.

Daniel Schwarz, Jan Fivaz und Alessia Neuroni beschäftigen sich in ihrem Beitrag „Die Haltung der Politik zu Digitalthemen mit Wirtschaftsbezug“ mit der Frage, wie Schweizer Politiker sich zu verschiedensten Aspekten der Digitalisierung positionieren. Befragt wurden Kandidierende anlässlich der nationalen Parlamentswahlen im Jahr 2019. Der Beitrag wertet gezielt digitalpolitische Fragen mit Wirtschaftsbezug aus und lässt ein differenziertes Meinungsbild erkennen, wenngleich auch bei einer Reihe von Digitalthemen der in der Tagespolitik weit verbreitete Links-Rechts-Gegensatz, z. B. beim Arbeitnehmerschutz und dem bedingungslosen Grundeinkommen, aufscheint. Es sind jedoch in vielen Themen aufgeweichte parteipolitische Fronten zu erkennen, dies gilt speziell auch für Forderung nach einem stärkeren staatlichen Engagement für berufliche Umschulungen oder diejenige nach einem internationalen Engagement der Schweiz zugunsten verbindlicher ethischer Leitlinien für den Einsatz von künstlicher Intelligenz. Dasselbe gilt für ein neues Grundrecht auf digitale Unversehrtheit (digitale Integrität), das in die Verfassung aufgenommen werden soll. Offen bleibt jedoch, ob das Ausmaß des Veränderungspotenzials von der Politik wirklich erkannt wird.

Mit den „Distributed Ledgers in der Energieversorgung“ legen Pascal Pfister und Jan Frèce eine Untersuchung vor, die das Potenzial von Blockchain für diverse Steuerungs- und Abwicklungsprozesse in der Energiewirtschaft untersucht. Dabei wird deutlich, dass deren Einsatz eine grundlegende Umstellung der Rollen und Prozesse im entsprechenden Wirtschaftsumfeld sowie der technologischen Grundlagen bedingt. Der Umbau induziert somit ein neues digitales Businessmodell und zeigt am Beispiel der Energieversorgung auf, welche Faktoren für eine mehrwert-generierende, sichere Implementierung der Ledger-Technologie berücksichtigt werden müssen: von technologischen Fragen bis zur Frage nach neuen Geschäftsmodellen und den damit verbundenen Risiken.

In ihrem Beitrag zu „Robotic Process Automation“ zeigen Hanka Arnautovic und Anja Habegger auf, wie in verschiedenen Branchen Prozessautomationen zum Einsatz kommen: dezentral gesteuerte, autonome Prozesse vernetzer Maschinen, Roboter, Werkstücke und Mitarbeitende z. B. für die Reduktion der Call-Zeit, der Steigerung des Umsatzes durch die Analyse von Störungsdaten oder durch den Einsatz eines Roboters zum Lesen von E-Mails. Robotic Process Automation stellt in dieser Entwicklung einen ersten Schritt auf dem Weg zu einer intelligenten Prozessautomation dar und bietet Unternehmen den Einstieg in die Digitalisierung und Automatisierung von Prozessen sowie in die Kollaboration von Menschen und Software-Robotern. Untersuchte Sektoren waren die Consulting-, Finanz-, Transport- und Versicherungsbranchen.

Die weiteren drei Beiträge können dem Bereich der Finanzen und digitalen Transformation zugeschrieben werden.

Der Beitrag von Patrik Graf, Markus A. Meier und Kim Oliver Tokarski widmet sich auch der „Robotic Process Automation“. Untersucht wird, wie Softwareroboter am Beispiel des Finanzbereichs der BKW AG zum Einsatz kommen. Die Resultate der Case Study zusammen mit den erhobenen Best Practices in den Unternehmen weisen das in der Theorie attestierte Nutzenpotenzial in der Praxis aus. Für die Implementierung von RPA ist ein Start mit einem motivierten Team sinnvoll, das erste Prozesse in einem Piloten automatisiert. Für die Verankerung im Unternehmen wird ein aktives Changemanagement sowie die frühzeitige Ausgestaltung des Operating Models empfohlen. Der Einsatz von RPA ist auch kein Selbstzweck, sondern bedarf einer sachlichen Grenznutzenabwägung.

Marco Birkhofer und Sandro Bächli haben mit ihrer Untersuchung zum „Open Banking und standardisierte Schnittstellen (API) auf dem Finanzplatz Schweiz“ Risiken und Chancen einer eigentlichen digitalen Transformation analysiert. Es zeigt sich, dass Open Banking in der Schweiz gegenüber den Vorreitern Großbritannien und der Europäischen Union noch wenig fortgeschritten ist. Durch innovative Services hat Open Banking das Potenzial, das bestehende Produkt- bzw. Serviceangebot zu erweitern und so die Customer-Experience der Kunden zu steigern. Hierzu wird empfohlen, etablierte und dabei geschlossene Geschäftsmodelle der Banken aufzubrechen, denn der Trend für die Zukunft deutet auf digitale Ökosysteme mit klarem Kundenfokus hin. Gleichwohl gibt es bei diesem neuen Ansatz auch Schattenseiten, denn offene Modelle können zu einer breiteren Angriffsfläche für Betrugsfälle führen. Um dem entgegenzuwirken, werden Sicherheits- und Compliancethemen immer bedeutsamer.

Im anschließenden Beitrag haben sich Michael Mathys und Raul Gimeno mit der „Integration von Kryptowährungen in das Angebot von Regionalbanken“ beschäftigt. Der Fokus der Untersuchung liegt bei der Einführung der Kryptowährungen auf Basis der zugrunde liegenden Blockchaintechnologie. Im Rahmen der Analyse wurden vier grundlegende Dienstleistungen identifiziert, welche auf Basis von Kryptowährungen angeboten werden können. Der erste Typ beinhaltet die Kryptowährung als Asset. Dabei können die verschiedenen Kryptowährungen als Wertanlage dienen. Im zweiten Typ wird Kryptowährung als Zahlungsmittel gesehen. Es handelt sich dabei um die Funktion eines digitalen Austauschs zum Bezug von Waren und Dienstleistungen. Darüber hinaus kann im Typ 3 die Begleitung eines Initial Coin Offering (ICO) und somit verstärkt die Kapitalbeschaffung für kleine und mittelgroße Unternehmen identifiziert werden. Zu guter Letzt können dem Typ 4 Kredite in Kryptowährungen und somit Finanzierungen in digitalen Währungen für Kunden mit Erträgen in Kryptowährungen zugeschrieben werden. Es wurde im Rahmen der analysierten Modellbank festgestellt, dass bis auf die Begleitung von ICO sämtliche Umsetzungen technische Anpassungen bedingen.

Die besondere Bedeutung des Faktors Mensch für einen erfolgreichen digitalen Wandel kommt in insgesamt vier Beiträgen zum Ausdruck.

Andrea Gurtner, Isabelle Clerc und Lena Scheidegger setzen sich in ihrem Beitrag zum „Digital Human Resource Management“ mit den Herausforderungen des Personalmanagements in der digitalen Transformation auseinander. Trotz hoher Relevanz der digitalen Transformation in den befragten Betrieben im Schweizer Mittelland wird die Qualifikation der Mitarbeitenden als noch nicht ausreichend angesehen. Zentrale Faktoren für die digitale Transformation in diesem Kontext sind ausgewählte Skills und spezifische persönlichkeitsbezogene Fähigkeiten, wie beispielsweise Offenheit und Flexibilität und ein damit verbundenes Mindset der Mitarbeitenden. Diese bedeutenden Faktoren sind eingebettet in eine entsprechende Organisationskultur. Flankierend sind fachliche Schulungen als Instrument der HRM-Transformation identifiziert worden. Das HRM ist aktuell meist noch stark mit der Digitalisierung der eigenen HR-Prozesse beschäftigt. Oft befindet sich das HRM somit noch in der ersten Phase der digitalen Transformation, der Digitization. Dies birgt die Gefahr, dass das HRM die Chance verpasst, als strategischer Partner die Entwicklungen im Unternehmen hin zu neuen digitalen und flexiblen Organisationskulturen mitgestalten zu können.

In „Employability 4.0“ zeigen Bruno Wymann und Jochen Schellinger, dass Veränderungen in der Arbeitswelt auch zu neuen Anforderungen an die Mitarbeitenden selbst und ihre individuelle Arbeitsmarktfähigkeit führen. Fallbasiert wurde für ein Schweizer Tochterunternehmen eines Industriekonzerns untersucht, welche bedeutenden Entwicklungen in der Schweizer Arbeitswelt zukünftig zu erwarten sind, wie sich diese auf die Arbeitsmarktfähigkeit der Mitarbeitenden auswirken und wie das HRM die Mitarbeitenden in der Weiterentwicklung ihrer Arbeitsmarktfähigkeit unterstützen kann. Als Ergebnis zeigt sich, dass Digitalisierung ein äußerst relevanter Megatrend in der Arbeitswelt ist. Vor diesem Hintergrund werden die Anpassungsfähigkeit sowie das lebenslange Lernen der Mitarbeitenden als die wichtigsten Kompetenzen zur Erhaltung und Förderung ihrer Arbeitsmarktfähigkeit identifiziert. Auf dieser Grundlage werden konkrete Vorschläge für die HR-Organisation und das HRM der untersuchten Unternehmensgruppe abgeleitet, wie zum Beispiel unterjährige Employability-Standortbestimmungen sowie die Entwicklung zielgerichteter Aus- und Weiterbildungsangebote.

Immanuel Zurbriggen und Jochen Schellinger entwickeln im Beitrag „Human Resource Management im Wandel der Digitalisierung“ Perspektiven cloudbasierter HR Shared Services (HRSS) für Klein- und mittelständische Unternehmen (KMU). Dabei wird als Ausgangsbasis ein hoher Nutzungsgrad an voll automatisierten, intelligenzbasierten und vernetzten HR-Systemen und Robotern postuliert. Hiermit verbunden ist eine starke Digitalisierung bzw. digitale Transformation von (administrativen) HR-Prozessen. Um dieser Annahme in der Praxis proaktiv begegnen und diese umsetzen zu können, wird ein für KMU einsetzbares, mehrwertgenerierendes Modell für cloudbasierte HR Shared Services entwickelt, gestaltungsbezogen validiert und optimiert. Ein wichtiges Kriterium eines solchen cloudbasierten HRSS sind Effizienzsteigerungen durch schnellere und effizientere HR-Prozesse. Als übergeordneter Mehrwert wird gleichermaßen die Schaffung von Freiraum für das HR-Kerngeschäft und dabei auch eine weitergehende Professionalisierung des HRM identifiziert.

In „Big Data: Konsequenzen für das Human Resource Management Schweizer Unternehmen“ nehmen Georg Reissich, Geraldine Rohr, Bernadette Wanzenried und Jochen Schellinger die Frage auf, wie große Datenmengen das HRM verändern. Der Wert von großen Daten (Big Data) und den hiermit verbundenen Informationen gilt als unbestritten, um beispielsweise neue Geschäftsmodelle oder neue Produkte zu entwickeln oder interne Prozesse zu optimieren. Für die Zukunft wird ein zunehmender Einsatz von Big-Data-Analysen auch im HRM vermutet, auch wenn derzeitige Anwendungen noch eher rudimentär erfolgen. Ein datenbasiertes Anwendungspotenzial wird aktuell vor allem im Gesundheitsmanagement und der Rekrutierung gesehen. Limitierende Faktoren der Ausnutzung von Big Data in der Anwendung sind rechtliche Restriktionen sowie ethische Bedenken von Entscheidungsträgerinnen und -trägern im HRM.

Schwerpunkt der nachfolgenden zwei Beiträge sind organisatorische und prozessuale Fragestellungen beim digitalen Change.

Sandra Odermatt und Eric Postler untersuchen am Beispiel des Paraplegiker Zentrums in Nottwil Formen der „Selbstorganisation als Enabler der Digitalisierung“. Selbstorganisation verspricht Lösungen für zentrale Führungsprobleme: Steigende Mitarbeiterzufriedenheit, höhere Produktivität und bessere Qualität durch kurze Entscheidungswege sowie geteilte Verantwortung sind nur einige davon. Ein dringender Handlungsbedarf im Gesundheitsweisen ergibt sich aus dem Fachkräftemangel, dem Kosten- und Leistungsdruck wie auch den starken Hierarchien. Für das Gesundheitswesen wird die Eignung der Selbstorganisation untersucht und es werden Handlungsempfehlungen für das Paraplegikerzentrum entwickelt. Dabei ist festzustellen, dass sich Selbstorganisation prinzipiell für alle Organisationen eignet. Von besonderer Bedeutung ist dabei die Schaffung eines gemeinsamen Verständnisses, die Initiierung und Realisierung eines Kulturwandels sowie die Implementierung eines neuen Führungsverständnisses.

Dominik Appius, Roger Andreas Probst und Kim Oliver Tokarski analysieren im „Beitrag Edge Computing und Industrie 4.0“ spezifische Anwendungsbereiche in der Schweizer Fertigungsindustrie. Durch die zunehmende immer höher werdende Vernetzung von Fertigungsanlagen im Rahmen der digitalen Transformation generiert die Schweizer Fertigungsindustrie als Konsequenz kontinuierlich hohe Datenmengen. Eine zielgerichtete Nutzung der Daten ohne lange Transportwege bei gleichzeitiger Verarbeitung dieser Daten zur Generierung und Nutzung neuen Wissens ist dabei eine zentrale Herausforderung des Edge Computing. Der vorliegende Beitrag liefert hierfür praxisnahe Erkenntnisse in den Bereichen technisches Verständnis, Geschäftsmodelle und Anwendungsszenarien sowie praktische Umsetzungen in Form von Pilotierungen und Roll-outs als Proof of Concept.

Vier weitere Beiträge fokussieren auf digitalisierungsbezogene Fragestellungen im Bereich des Marketings.

Im Beitrag „Schlüsselfaktoren im Marketingkonzept von Schweizer Gig-Workern“ von Tobias Burri und Etienne J. Rumo wird ein anwendungsorientiertes Grundwissen für die Erarbeitung eines Marketingkonzepts von Gig-Workern präsentiert. Dabei sind Marketingmixrichtlinien erarbeitet worden, die als Ausgangspunkt und Grundeinstellung individueller Konzeptentwicklungen verwendet werden können. Auf der Basis handlungsorientierter Leitfragen können individuelle Antworten erarbeitet werden, welche den Gig-Workern die zu verwendenden Kanäle, Maßnahmen, Instrumente und Inhalte für das persönliche Marketingkonzept aufzeigen.

Livia Kernen, Benjamin Adriaensen und Kim Oliver Tokarski präsentieren im Beitrag „Social Influencer“ eine quantitative Analyse erfolgreicher Instagram-Influencer. Als wichtiges Ergebnis zeigt sich, dass Glaubwürdigkeit bzw. Authentizität der bedeutendste Erfolgsfaktor für Social Influencer ist. Weitere bedeutende Faktoren sind die persönliche Beziehung sowie die Interaktion mit der Community. Auf der Aktionsebene ist festzustellen, dass die analysierten Social Influencer zu spezifischen Zeiten ihre Beiträge posten, in denen sie durchschnittlich am meisten Likes und Kommentare generieren können. Dabei sind nicht nur Bilder wichtig, denn es werden auch viele Bildunterschriften, jedoch wenige Hashtags, verwendet. Beiträge mit „freizügigen“ Inhalten erzeugen durchschnittlich am meisten Likes und Kommentare. Durch die persönliche Beziehung zur Community geben die Social Influencer einen „Einblick in ihr Leben“. So zeigen die Analysen, dass Bilder der Social Influencer selbst oder Beiträge mit speziellen Ereignissen aus ihrem Leben tendenziell am meisten Likes erhalten. Oft wird dabei ein positives Bild generiert. Denn bei den analysierten Social Influencern lassen sich fast keine Beiträge mit gewalttätigen, betrügerischen oder medizinischen Inhalten aufweisen.

Im Beitrag „Datenbasierte Weiterentwicklung des Kundenerlebnisses in der Möbelbranche“ zeigen David Aemmer, Jonas Bigler und Deane Harder, dass das Konsumentenverhalten beim Kauf von Möbeln relativ komplex ist. Es wird dabei im Sinne einer Customer Journey aufgezeigt, wie Kunden beim Möbelkauf vorgehen, welche Kanäle sie verwenden und welchen Herausforderungen sie begegnen. Es zeigt sich, dass sich Kunden oftmals zuerst online über ein Möbelstück informieren. Aufgrund von fehlenden, aber für den Kauf relevanten Informationen wird die Customer Journey im stationären Handel weitergeführt. Für die Möbelbranche stellt sich die Frage, wie der Kaufentscheid online erleichtert, der Kanalwechsel besser gestaltet und der Anschluss an die Onlinerecherche nahtlos gewährleistet werden kann. Ein bedürfnisorientierter Zugang zu Produkten, auf Kundendaten basierende Produktempfehlungen, verknüpfte Kanäle sowie eine transparente und glaubwürdige Datenstrategie sind hierbei entscheidende Erfolgsfaktoren.

„Mit Nudging zu mehr Spenden auf Webseiten anregen“: Lukas Keller und Deane Harder fragen in ihrem Beitrag, ob Soziale-Norm-Nudges die Bereitschaft für Onlinespenden auf Schweizer Webseiten von Nonprofitorganisationen erhöhen können, denn der Spendenmarkt ist weitgehend gesättigt und durch die wachsende Anzahl an Stiftungen und Vereinen wächst der Wettbewerb immer weiter. Für Nonprofitorganisationen stellt sich die Herausforderung, wie ihr Fundraising weiterentwickelt werden kann. Eine Möglichkeit ist die Nutzung des Potenzials von Onlinespenden. Vor diesem Hintergrund untersucht der Beitrag, ob Soziale-Norm-Nudges zur Erhöhung der Spendenbereitschaft auf Schweizer Webseiten von NPO beitragen.

Alle Beiträge des Herausgeberwerks lassen sich den Maturitätsstufen des Analysemodells für Digital Business und digitale Transformation zuordnen. Es zeigt sich dabei eine hohe Bandbreite an Themen und Praxisanwendungen im Sinne von Modellen, Analysen und Handlungsfeldern über alle Stufen hinweg.
